# *Cryptococcus gattii* Complex Infections in HIV-Infected Patients, Southeastern United States

**DOI:** 10.3201/eid2411.180787

**Published:** 2018-11

**Authors:** Kaylee T. Bruner, Carlos Franco-Paredes, Andrés F. Henao-Martínez, Gregory M. Steele, Daniel B. Chastain

**Affiliations:** Phoebe Putney Memorial Hospital, Albany, Georgia, USA (K.T. Bruner, G.M. Steele, D.B. Chastain);; Hospital Infantil de Mexico, Federico Gomez, Mexico City, Mexico (C. Franco-Paredes);; University of Colorado Denver Anschutz Medical Campus, Aurora, Colorado, USA (C. Franco-Paredes, A.F. Henao-Martínez);; University of Georgia College of Pharmacy, Albany (D.B. Chastain)

**Keywords:** Cryptococcus, Cryptococcus gattii, Cryptococcus gattii complex, fungi, meningitis/encephalitis, infections, HIV/AIDS and other retroviruses, virus, HIV-infected patients, case series, southwestern Georgia, southeastern United States, Pacific Northwest, United States

## Abstract

*Cryptococcus gattii* traditionally infects immunocompetent hosts and causes devastating pulmonary or central nervous system disease. However, this infection rarely occurs in patients infected with HIV. We report 3 cases of HIV-associated *C*. *gattii* complex infections in the southeastern United States. Detection of *C*. *gattii* in HIV-infected patients in this region warrants increased awareness of this threat to ensure appropriate diagnosis and treatment to optimize patient outcomes.

## Abstract 2

Increased awareness of *C. gattii* infections in these patients is critical for improving diagnosis, treatment, and outcomes.

*Cryptococcus gattii* is an encapsulated fungus found primarily in tropical and subtropical regions such as Australia and South America (*1*). *C*. *gattii* was first documented as an emerging pathogen in the United States in 1999 when an outbreak occurred in Oregon and Washington (*1*). More recently, sporadic cases have been reported throughout the southeastern United States (*2**–**4*). Theories for the emergence of *C. gattii* in temperate areas have included climate change, increased travel, and anthropogenic activity; however, the exact mechanism remains unknown (*1**,**4*).

*C. gattii* was previously known as variant of *C. neoformans* but was later recognized as an independent species of *Cryptococcus* (*4*). Recently, *C*. *gattii* was reclassified as a species complex comprised of 4 individual species (*5*). Similar to *C. neoformans*, *C. gattii* is acquired through inhalation; infection can progress to pneumonia and central nervous system disease by dissemination into the bloodstream. *C. gattii* has been associated with increased virulence and more severe neurologic manifestations than *C. neoformans*, resulting in major illness and death (*3*). Although treatment is currently the same as for *C. neoformans*, it has previously been suggested that more aggressive management of neurologic complications, including decreasing elevated intracranial pressure and possibly early use of dexamethasone, might be warranted for *C. gattii* cases (*2**, **3*). In addition, longer duration of induction therapy, averaging approximately 6 weeks, has been required for *C. gattii* infections (*6*).

*C. gattii* infection has traditionally been reported more often in immunocompetent persons, in contrast to *C. neoformans,* which is more prominent in severely immunocompromised hosts, particularly among those with HIV/AIDS (*3**,**7*). However, more recent evidence has identified some potential risk factors for *C. gattii* meningoencephalitis. These factors include antibodies against granulocyte–macrophage colony-stimulating factor, which leads to macrophage dysfunction, and chronic medical conditions, including diabetes mellitus and other illnesses, such as end-stage liver or renal disease (*8**–**10*).

Cases of *C. gattii* meningoencephalitis in HIV-infected patients have been reported rarely in areas with high HIV prevalence, such as Botswana and sub-Saharan Africa (*11*). It appears that the only cases reported of *C. gattii* in HIV/AIDS patients in the United States have been limited to a small number in southern California (*12*).

We report 3 cases of *C. gattii* complex meningitis and pneumonitis in HIV-infected patients residing in southwestern Georgia. These cases should alert clinicians for detection of HIV-associated *C. gattii* complex in the southeastern United States.

## Case-Patient 1

A 34-year-old man with a history of infection with HIV and medication noncompliance was admitted to Phoebe Putney Memorial Hospital (Albany, GA, USA) because of a 5-week history of nausea, vomiting, and weight loss. He also had headaches, photophobia, and subjective syncope. The patient had a CD4+ T-cell count of 6 cells/mm^3^ and an HIV-1 RNA level of 71,265 copies/mL. He reported no recent travel history or exposure to animals. At admission, initial workup included a barium swallow procedure and kidney, ureter, and bladder radiography. These procedures showed no unusual findings.

After we observed an additional syncopal episode, we ordered a test for serum cryptococcal antigen (CrAg) and magnetic resonance imaging (MRI) of the brain because of the HIV status of the patient and concern for an intracranial infectious process. After detection of a serum CrAg titer >1:2,560, a lumbar puncture (LP) was performed on day 4 of hospitalization. The LP showed an opening pressure of 24 cm of water, 5 leukocytes/mm^3^ (6% polymorphonuclear cells and 94% mononuclear cells), 0 erythrocytes/mm^3^, a protein level of 29 mg/dL, and a glucose level of 49 mg/dL. A positive result (titer >1:2,560) was observed for CrAg in cerebrospinal fluid (CSF).

The patient was given intravenous (IV) amphotericin B lipid complex (5 mg/kg/d) and oral flucytosine (25 mg/kg 4×/d). On day 5, a repeat LP was performed to evaluate intracranial pressure and showed identical opening and closing pressures of 5 cm of water. After 5 days of treatment with amphotericin B lipid complex and flucytosine, renal dysfunction and thrombocytopenia developed on hospital day 9. The patient was then given oral fluconazole (800 mg 1×/d). Blood and CSF cultures grew *Cryptococcus* sp., which we further identified as *C. gattii* complex by using matrix-assisted laser desorption/ionization-time of flight mass spectrometry.

MRI of the brain showed enhancement of right frontal lobe adjacent to the lateral ventricle with subtle nodular enhancement within the right caudate head. Nonenhancing T2 and fluid-attenuated inversion recovery MRI showed hyperintensities within bilateral deep nuclei.

After 14 days of antifungal therapy, the patient was deemed stable. He was discharged and received oral fluconazole (800 mg 1×/d). He was scheduled for follow-up in the outpatient clinic 2 weeks later for a repeat LP and initiation of antiretroviral therapy (ART). Unfortunately, the patient did not return for continued care.

## Case-Patient 2

A 47-year-old man with a medical history of hypertension and infection with HIV was admitted to Phoebe Putney Memorial Hospital because of a 2-week history of fever, nausea, headaches, and unsteady gait. Outpatient records showed a CD4+ T-cell count of <20 cells/mm^3^ and an HIV-1 RNA level of 1,653 copies/mL, for which he was recently given ART. This therapy consisted of emtricitabine (200 mg 1×/d), tenofovir disoproxil fumarate (300 mg 1×/d), raltegravir (400 mg 2×/d), and etravirine (200 mg 2×/d).

MRI of the brain performed at admission was unremarkable, with no definitive evidence of acute ischemic, intracranial hematoma, or enhancing intracranial lesion. Initially, the patient was given levofloxacin for treatment of possible sinusitis, but he continued to experience intermittent episodes of fever and persistent headaches. On day 2 after admission, an LP was performed and showed increased opening pressure, 85 leukocytes/mm^3^ (1% polymorphonuclear cells and 99% mononuclear cells), 11 erythrocytes/mm^3^, a protein level of 96 mg/dL, and glucose level of 42 mg/dL. A positive result (titer >1:256) was observed for CrAg in CSF.

The positive finding for CrAg prompted initiation of induction therapy for cryptococcal meningitis, which consisted of IV liposomal amphotericin B (5 mg/kg/d) and oral flucytosine (25 mg/kg 4×/d). CSF cultures grew yeast, which we eventually identified as *C. gattii* complex by using l-canavanine, glycine, bromothymol blue (CGB) agar. Blood cultures remained sterile throughout hospitalization. The patient was given IV dexamethasone (4 mg every 6 h) because of recurrent headaches. Before completion of 14 days of induction therapy, a repeat LP was performed and showed an opening pressure of 43 cm of water, a closing pressure of 30 cm of water, 97 leukocytes/mm^3^ (100% mononuclear cells), 4 erythrocytes/mm^3^, a protein level of 89 mg/dL, and a glucose level of 58 mg/dL. CSF remained positive for CrAg, but the titer decreased to 1:16, and the CSF culture remained sterile.

The patient was discharged and received voriconazole and a dexamethasone taper over a 6-week period. Voriconazole was continued for 6 months and was chosen for the consolidation phase of treatment because there is some evidence that the MIC of voriconazole is lower than the MIC of fluconazole for most *C. gattii* complex isolates (*13*,*14*). During the subsequent 5 years of follow-up, his ART was changed to abacavir (300 mg 2×/d), lamivudine (150 mg 2×/d), and raltegravir (400 mg 2×/d). The patient continued secondary prophylaxis with oral voriconazole until CD4+ T-cell counts were >100 cells/mm^3^ for at least 3 months. Recent laboratory results showed undetectable HIV-1 RNA and a CD4+ T-cell count of 256 cells/mm^3^.

## Case-Patient 3

A 47-year-old man with a history of depression and infection with HIV for ≈20 years was admitted to Phoebe Putney Memorial Hospital because of suicidal ideation caused by persistent pain. He also reported body aches, fatigue, and weakness for a 1-month duration, but denied pulmonary symptoms or fevers. He was previously receiving ART, but had been noncompliant for the previous 2 years because of homelessness.

At admission, initial examination showed a CD4+ T-cell count of 26 cells/mm^3^, an HIV-1 RNA level of 272,152 copies/mL, positive result for serum CrAg (titer 1:160), and a right hilar mass by chest radiograph. The patient was given IV amphotericin B lipid complex (5 mg/kg/d) and oral flucytosine (25 mg/kg 4×/d). An LP showed 3 leukocytes/mm^3^ (100% mononuclear cells), 4 erythrocytes/mm^3^, a protein level of 42 mg/dL, and a glucose level of 62 mg/dL. The patient had a negative result for CrAg in CSF. 

Because of these findings, we performed computed tomography (CT) of the chest and identified a mass (4.0 cm × 2.5 cm) with central cavitation in the right lower lobe abutting the pleural surface (Figure). The patient underwent a CT-guided lung biopsy to evaluate the right lower lobe mass. Pathologic examination demonstrated fibrous tissue and numerous cystic spaces containing macrophages and variably sized organisms with a thick capsule that were strongly positive when stained with Grocott’s methanamine silver special stain. The morphologic features were consistent with those of a *Cryptococcus* sp. We observed many budding yeasts by using Gram stain.

**Figure:**
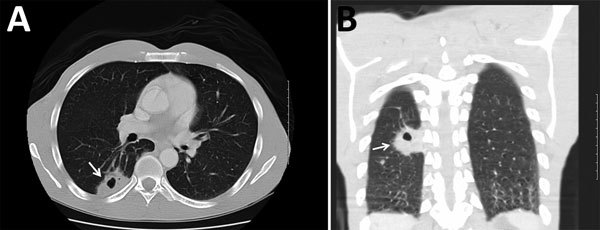
Computed tomography images of the chest of an HIV-infected 47-year-old man (case-patient 3) with *Cryptococcus gattii* complex infection, southeastern United States. Transverse (A) and frontal (B) views without intravenous contrast showed a mass (arrows) (4.0 cm × 2.5 cm) that had central cavitation posteriorly in the right lower lobe abutting the pleural surface. The central cavitary portion of this lesion had a maximum length of ≈1.3 cm and no evidence of fluid level or internal soft tissues

The patient continued to receive oral flucytosine (25 mg/kg 4×/d), but because of an acute kidney injury after 3 days, amphotericin B lipid complex was replaced with IV fluconazole (800 mg in a single dose), followed by oral fluconazole (400 mg 1×/d). He remained hospitalized for an additional 3 days until growth on CGB agar was identified as *C*. *gattii* complex. At that time, the patient had improved considerably and was discharged.

During outpatient treatment, the patient received emtricitabine (200 mg/d), dolutegravir (50 mg/d), darunavir (800 mg/d), and cobicistat (150 mg/d). A CT of his abdomen and pelvis to evaluate lower abdominal pain was performed ≈6 months later and showed resolution of the initial mass. We detected a new 20-mm lesion within the right lower lobe, which was not observed in previous studies. The patient continued to receive fluconazole (400 mg 1×/d) for an additional 12 months without subsequent radiographic monitoring. His most recent laboratory results showed a CD4+ T-cell count of 250 cells/mm^3^ and an HIV-1 RNA level <40 copies/mL while receiving the aforementioned ART.

## Discussion

We report 3 cases of *C. gattii* complex infections in HIV-infected patients in the southeastern United States (southwestern Georgia). Given the increasing recognition of *C. gattii* complex in southwestern Georgia, it is routine practice at our institution to plate cryptococcal isolates on CGB agar to distinguish between *C. neoformans* and *C. gattii* complex (*4**,**15*). Since implementation of this practice in 2012, of 30 clinical isolates of *Cryptococcus* sp., 3 (10%) have been identified as the *C*. *gattii* complex. Although the environmental source of these infections in southwestern Georgia remains unknown because the 3 patients in this case series had no previous travel, we believe that their immunocompromised state characterized by low CD4+ T-cell counts is a contributing factor for acquiring infection with *C. gattii* complex.

Historically, most cases of infection with *C. gattii* were identified in patients who were immunocompetent or otherwise healthy and lived in the Pacific Northwest region of the United States because of endemicity of this fungi to this region (*3*). However, recent data suggest a longer duration of endemicity in the southeastern United States than in the Pacific Northwest, specifically because of the *C*. *gattii* VGI-SE clade (*16**,**17*).

Data from the 1960s and 1980s indicated detection of *C*. *neoformans* serotypes B and C, now recognized as *C*. *gattii*, from clinical and environmental isolates. However, the first cases of *C*. *gattii* infections from regions outside of the Pacific Northwest were reported in 2009. In a retrospective database review by the Centers for Disease Control and Prevention for 2004–2011, a total of 96 case-patients infected with *C. gattii* were identified; 81 resided in the Pacific Northwest (Washington and Oregon) and most were infected with the VGII outbreak strain, which was acquired from the outbreak on Vancouver Island in Canada (*10*). Nonoutbreak strains VGI and VGIII were confined to HIV-negative persons residing in states outside the Pacific Northwest in comparison with outbreak case-patients, of whom 3 were infected with HIV. Similar to the findings for case-patient 3 in our case report, 59% of patients in the Pacific Northwest outbreak had *C. gattii* pneumonia; however, 75% had respiratory symptoms, but case-patient 3 had no respiratory symptoms (*10*). 

In 2010, 276 isolates of *Cryptococcus* sp. in HIV-infected patients were reported in southern California, of which 34 isolates were identified as *C. gattii*, demonstrating that *C. gattii* had been present previously in immunocompromised patients (*12*). More recently, 25 case-patients outside the Pacific Northwest have been reported; 5 had *C. gattii* pulmonary disease, 12 had central nervous system disease, and 8 had a combination of the 2 *C. gattii* diseases (*2*). Of the 25 case-patients reported, 7 resided in the southeastern states: 1 in Florida, 5 in Georgia, and 1 in Alabama. The HIV status of these case-patients was not reported; however, the case-patient in Florida was an otherwise healthy native of Florida who had no known travel to areas to which *C. gattii* is endemic (*18*). Similar to case-patients 1 and 2, two previous case-patients with *C. gattii* meningitis were reported in southwestern Georgia (*3**,**15*). The surprising difference was that these patients were HIV negative and otherwise healthy.

Speciation of cryptococcal isolates is essential because *C. gattii* complex meningoencephalitis might have more severe clinical manifestations requiring aggressive management of intracranial hypertension with early evacuation of CSF and treatment with steroids (*3*). Use of dexamethasone for treatment of *C. gattii* complex meningitis has been associated with positive outcomes by potentially decreasing cerebral edema and tissue damage (*19**,**20*). In a small retrospective study of 16 patients in Papua New Guinea infected with *C. gattii*, most patients who received corticosteroids had reduced rates of vision loss and blindness (*21*). In addition, case-patients infected with the *C. gattii* complex might require prolonged courses of amphotericin B–based therapy compared with case-patients infected with *C. neoformans* (*6*). Finally, there appears to be different antifungal susceptibility to azoles that might suggest the use of voriconazole instead of fluconazole for the consolidation phase of antifungal therapy (*13**,**14*).

Early identification of the *C. gattii* complex can be challenging because of limited accessibility of tests that differentiate *Cryptococcus* spp., including use of CGB agar (*22*). Because of lack of availability of testing in local microbiology laboratories, it is likely that infection with *C. gattii* complex is underreported.

On the basis of the cases presented in this report and continued detection of *C*. *gattii* in the southeastern United States, we believe routine testing to differentiate *C*. *neoformans* from *C*. *gattii* is warranted and further investigation is required to determine appropriate treatment in comparison with treatment for *C. neoformans*. In addition, healthcare providers should be aware of this pathogen and its potential to cause devastating disease in immunocompromised and immunocompetent patients.
